# The Enterics for Global Health (EFGH) *Shigella* Surveillance Study in Mali

**DOI:** 10.1093/ofid/ofae003

**Published:** 2024-03-25

**Authors:** Adama Mamby Keita, Sanogo Doh, Jane Juma, Dilruba Nasrin, Awa Traoré, Uma Onwuchekwa, Rebecca Maguire, Fadima C Haidara, Samba O Sow, Karen L Kotloff, Milagritos D Tapia

**Affiliations:** Centre pour le Développement des Vaccins du Mali (CVD-Mali), Bamako, Mali; Centre pour le Développement des Vaccins du Mali (CVD-Mali), Bamako, Mali; Centre pour le Développement des Vaccins du Mali (CVD-Mali), Bamako, Mali; Center for Vaccine Development and Global Health, University of Maryland School of Medicine, Baltimore, Maryland, USA; Department of Medicine, University of Maryland School of Medicine, Baltimore, Maryland, USA; Centre pour le Développement des Vaccins du Mali (CVD-Mali), Bamako, Mali; Centre pour le Développement des Vaccins du Mali (CVD-Mali), Bamako, Mali; Center for Vaccine Development and Global Health, University of Maryland School of Medicine, Baltimore, Maryland, USA; Centre pour le Développement des Vaccins du Mali (CVD-Mali), Bamako, Mali; Centre pour le Développement des Vaccins du Mali (CVD-Mali), Bamako, Mali; Center for Vaccine Development and Global Health, University of Maryland School of Medicine, Baltimore, Maryland, USA; Department of Pediatrics, University of Maryland School of Medicine, Baltimore, Maryland, USA; Department of Medicine, University of Maryland School of Medicine, Baltimore, Maryland, USA; Center for Vaccine Development and Global Health, University of Maryland School of Medicine, Baltimore, Maryland, USA; Department of Pediatrics, University of Maryland School of Medicine, Baltimore, Maryland, USA; Department of Medicine, University of Maryland School of Medicine, Baltimore, Maryland, USA

**Keywords:** diarrhea, global health, Mali, *Shigella*, surveillance

## Abstract

**Background:**

In 2002, the Centre pour le Développement des Vaccins du Mali (CVD-Mali) was established as a partnership between the Mali Ministry of Health and the University of Maryland, Baltimore. Since its creation, CVD-Mali has been dedicated to describing the epidemiology of infectious diseases, supporting the development of vaccines, and training a team of local researchers. CVD-Mali participated in the Global Enteric Multicenter Study from 2007 to 2010 and the Vaccine Impact on Diarrhea in Africa study from 2015 to 2018, where the importance of *Shigella* as an enteric pathogen was established.

**Methods:**

In the Enterics for Global Health (EFGH) *Shigella* surveillance study, CVD-Mali will conduct *Shigella* surveillance at 4 health centers serving the population currently participating in a demographic surveillance system and will measure the local incidence of *Shigella* diarrhea and related outcomes in 6- to 35-month-old children. Antibiotic sensitivity patterns and the costs related to these cases will also be measured.

**Results:**

We anticipate reporting the number of diarrhea episodes that are positive by stool culture, the antibiotic susceptibility of these isolates, and the management and outcomes of these cases.

**Conclusions:**

In Mali, the EFGH study will contribute valuable information to understanding the burden of *Shigella* in this population. These data will inform the evaluation of vaccine candidates.

## Teaser

CVD-Mali has developed an infrastructure that enables detailed investigations of diarrheal diseases among infants and young children in Mali to inform the development of effective interventions. The results from EFGH will inform the conduct trials with promising vaccine candidates.

## SITE DESCRIPTION

In 2002, the Centre pour le Développement des Vaccins du Mali (CVD-Mali) was established as a partnership between the Mali Ministry of Health and the University of Maryland, Baltimore. CVD-Mali is a legal government entity working under the Ministry of Health. Since its creation, CVD-Mali has been dedicated to describing the epidemiology of infectious diseases, supporting the development of vaccines, and training a team of local researchers.

Mali is a largely arid country with daily average temperatures ranging from 20°C to 40°C. The climate in Bamako, the capital city, is characterized by distinct rainy and dry seasons. The rainy season, which lasts from May to October, is hot and cloudy, and there is a >45% chance that any day during this period will have precipitation. August has the rainiest days in Bamako, with an average of 27.4 days with precipitation. In contrast, the dry season lasts >7 months, from October to May, with December being the month with the least rain in Bamako [1].

The hottest months of the year are March through May, with an average daily temperature >30°C. The hottest month in Bamako is April, with an average maximum temperature of 40°C and a minimum of 28°C. The coldest month of the year in Bamako is January, with an average minimum of 15°C.

The Gross Domestic Product (GDP) per capita in 2021 was US$873 [2]. Undernutrition remains a significant public health issue, with 9.3% of under-5 children suffering from wasting and 19.4% experiencing stunted growth in 2021 [3]. Additionally, the level of maternal education is low, with 38.5% of women aged 15–24 years being illiterate in 2020 [4]. Access to water and sanitation facilities is limited, with only 76% having access to improved or adequate water and 59% having access to sanitation facilities [5]. No significant improvement had been observed as of 2021 [6]; therefore, the government has committed to providing everyone in Mali with clean water, decent toilets, and good hygiene by 2030 [7].

In 2006, CVD-Mali established a demographic surveillance system (DSS) in 2 neighborhoods in Bamako. One neighborhood, Banconi, is situated in Commune 1 and is in the eastern part of the city, north of the Niger River, and occupies an area of 6.23 km^2^. In 2022, the population was estimated at 153 490, with 14 556 <3 years of age. The second neighborhood is Djikoroni-para; it has a surface area of ∼5 km^2^. It is situated in Commune 4 and is in the western part of Bamako. The estimated population was 82 422 in 2022, with 7407 <3 years of age. These areas are among the most densely populated in the city. The population of the DSS quartiers in Bamako largely subsists on small business activity, trading, foreign remittances, and government employment [8].

Since its inception, an average of 2 rounds of demographic data collection have been conducted annually as part of the DSS. The Global Enteric Multicenter Study (GEMS; 2007–2010) and Vaccine Impact on Diarrhea in Africa (VIDA; 2015–2018) studies utilized this DSS to conduct community household surveys to understand health care–seeking patterns of young children and to extrapolate etiologic determinations among children seeking care for diarrheal diseases at health centers serving the DSS to derive population-based incidence estimates [9–11]. A baseline cross-sectional Healthcare Utilisation and Attitudes Survey (HUAS) was conducted before starting GEMS from March 9 to May 12, 2007, followed by a series of brief HUAS-lite surveys conducted during GEMS from January 2009 to March 2011 [5]. Repeated Healthcare Utilization and Coverage Surveys (HUCS) were conducted at least twice a year for the 36-month VIDA study period, between May 2015 and July 2018. Marked socioeconomic improvement was observed in the DSS area between GEMS and VIDA, with an increase in primary caretaker's education (completed primary school, 17.1% in GEMS vs 43.4% in VIDA) and a marked increase in assets for households (electricity: 73.8% vs 98.5%; television: 67.5% vs 94.4%; telephone: 86.4% vs 98.7%; motor/scooter: 60.5% vs 83.4%) (Unpublished data). Of note, the prevalence of diarrhea decreased (13.3% in GEMS vs 2.2% in VIDA), and care-seeking at a sentinel health center also decreased (11.5% in GEMS vs 8.3% in VIDA).

**Figure 1.**
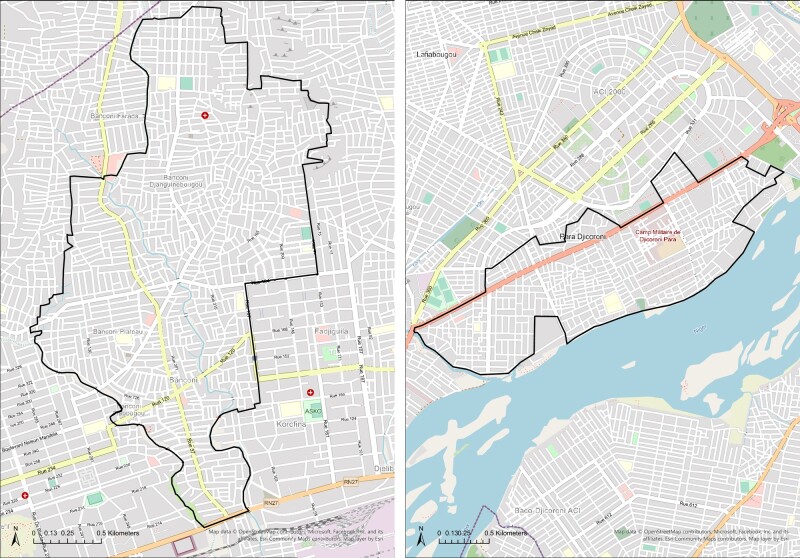
EFGH study catchment area—Banconi and Djikoroni-para, Bamako, Mali.

The EFGH study will be conducted in the same DSS catchment area as was used in GEMS and VIDA (Figure 1) at 4 of the 6 health centers previously used. These health centers serve the catchment population in the DSS area and comprise both Centres de Sante Communautaire (CSCOMs), which are the community health centers, and district health centers (CSREF). In Banconi, we will be working at the local CSCOM (Community Health Association of Banconi [ASACOBA]), and in Djikoroni-para, the study personnel will enroll at the Community Health Association of Djenekabougou (ASACODJENEKA) and Djikoroni-para (ASACODJIP). The fourth center is the district-level referral center in Banconi, CSREF Commune 1. These 4 health centers were selected based on their functionality and their successful participation in the GEMS and VIDA studies. Currently, each health center performs from ∼10 000 to ∼70 000 pediatric outpatient visits each year, and common diagnoses include gastroenteritis and pneumonia. GEMS and VIDA also enrolled children from the DSS population who were seeking care at the major pediatric referral hospital in Bamako, but this will not be done in EFGH.

The CSCOMs offer only outpatient services including ante/postnatal care, labor and delivery services, malnutrition management, and vaccinations. The infrastructure comprises the administrative unit led by the chief medical officer, the outpatient visits offices, a small lab that performs basic tests, the payment office, the maternity ward, and the pharmacy.

The CSREFs offer a higher level of care, with both outpatient and inpatient services (adult, pediatric, and neonatal unit), ante/postnatal care and labor and delivery services (including cesarean section), a vaccination program, laboratory capacity to conduct blood cell count and basic immunology tests, and access to general practitioners, obstetricians, pediatricians, pharmacists, and other providers. In addition to the infrastructure provided at a CSCOM, the CSREF has an operating room, which is used mainly for C-section and minor surgical procedures, pediatric unit, otolaryngology office, tuberculosis management office, HIV management office, immunization office, health insurance office, storage, and the morgue.

Mandatory health insurance (Assurance Médicale Obligatoire [AMO]) has been implemented by the government. Formal employees (public and private) are covered by this insurance in addition to a few of those self-employed. This insurance pays for two-thirds of hospitalization costs and prescriptions. AMO is not available at all CSCOMs. Therefore, people prefer to go directly to facilities where the AMO service is available.

## MANAGEMENT OF DIARRHEA

Management of diarrhea at these health facilities is determined by the treating physician. In both GEMS and VIDA, funding was provided for procurement of Integrated Management of Childhood Illness (IMCI)–recommended treatment of diarrhea, such as oral rehydration solutions (ORS) and antibiotics; however, clinical decision-making was the responsibility of the health facility staff. While care is supposed to be provided in line with the local guidelines, which largely align with the IMCI recommendations provided by the World Health Organization (WHO) [12], comparing the practices observed for treatment of moderate to severe diarrhea during GEMS and VIDA has shown that adherence to IMCI case management guidelines for children aged <5 years may be suboptimal.

Children who had no dehydration when evaluated at the health center saw an increase from 41.9% to 68.8% between GEMS and VIDA in the percentage who were offered more fluids than usual at home, while the percentage with continued feeding (59.3% vs 56.2%) remained similar [13]. Although oral rehydration was administered in the health care facility to <5% of children with either no, some, or severe dehydration, nearly all children were given a prescription for ORS to be taken at home. Intravenous rehydration was given to only 27.8% and 20.5% of children with severe dehydration in GEMS and VIDA, respectively. Fewer than 15% of children in VIDA were prescribed zinc, although this was an increase compared with GEMS, which occurred before zinc rollout in Mali.

Antibiotics were commonly prescribed for children in VIDA who did have an IMCI indication—such as dysentery, suspected cholera, or a clinical diagnosis such as pneumonia, meningitis, or other invasive bacterial infection, otitis media, tonsillitis, or pharyngitis—or severe acute malnutrition [14]. The most prescribed antibiotics for dysentery in VIDA were metronidazole (83.3%) and cotrimoxazole (68.3%), neither of which is IMCI-recommended; moreover, 80% of *Shigella* isolates in Mali were cotrimoxazole-resistant [15].

In EFGH, to encourage the health facility clinicians to manage diarrhea according to national standards, the study team will review the management, giving feedback to the treating physician and providing medications as needed per the national guidelines, which largely follow WHO IMCI guidelines (Table 1) [16]. The guidelines address the management of dehydration, with and without severe acute malnutrition and zinc and antibiotic administration. Dehydration management is stratified by severity so that the most severely dehydrated patients are administered intravenous fluids, whereas those with no dehydration are encouraged to drink oral rehydration solution in greater-than-usual quantities. Moreover, patients with concurrent severe malnutrition are recommended ReSoMal, which is currently available in Mali. Patients with some or severe dehydration should be reassessed after being treated at the health facility. Zinc is prescribed for all children, irrespective of hydration and nutrition status. Antibiotics are recommended for children who present with severe malnutrition, dysentery, or have a stool culture positive for *Shigella*. The recommended antibiotic for treatment of *Shigella* is azithromycin, ciprofloxacin, or ceftriaxone.

**Table 1.** Diarrhea Management Guidelines According to the Mali Ministry of Health

**Dehydration Management (without Severe Acute Malnutrition)**		
Dehydration level	WHO-Indicated Management	Mali-Specific Guideline
Severe	Plan C Start IV fluid immediately (preferably ringers Lactate Solution [100 mL/kg]) Reassess every 15–30 min Give ORS (5 mL/kg/h) as soon as the child can drink Reclassify dehydration after 6 h in infant & 3 h in child and continue with A, B, C plan	Ringers Lactate Solution by IV (100 mL/kg) for 6 h in infant & 3 h in child Reassess and adapt the treatment Zinc Antisecretory (dio-smectite) Anti-emetic (vogalene) Antibiotic (see below)
Some	Plan B Give recommended ORS in clinic over 4 h Reclassify dehydration after 4 h and continue with A, B, C plan	Give recommended ORS in clinic over 4 h Reclassify dehydration after 4 h and adapt the treatment Give zinc as in Plan A
None	Plan A Increase food and fluid intake to prevent dehydration	More fluid than usual: ORS Zinc Food Mother education (when to come back)
**Dehydration Management (Severe Acute Malnutrition & No Shock)**		
Dehydration level	WHO-Indicated Management	Mali-Specific Guideline
Severe	Plan C Give ReSoMal^a^ (or half-strength standard low ORS with added potassium & glucose) 5 mL/kg every 30 min for the first 2 h 5–10 mL/kg per h for the next 4–10 h on alternate hours with F75 If rehydration still required at 10 h, give starter F-75 instead of ReSoMal at the same times	ReSoMal 5 mL/kg every 1 h
Some	Plan B Same as above	Same as above
^a^In child with suspected cholera, use standard ORS		
**Zinc**		
Population	WHO-Indicated Treatment	Mali-Specific Guideline
All children	Zinc supplementation for 10–14 d ≤6 m 10 mg per day >6 m 20 mg per day	Zinc supplementation for 14 d ≤6 m ½ tablet per day >6 m 1 tablet per day
**Antibiotics**		
Population	WHO-Indicated Treatment	Mali-Specific Guideline
Dysentery or *Shigella* upon culture confirmation	Ciprofloxacin (15 mg/kg) twice daily for 3 d OR based on local sensitivity IV/IM Ceftriaxone at 50–80 mg/kg per day for 3 d (if child is severely ill or as second-line treatment) Other second-line therapies (azithromycin, cefixime, trimethoprim- sulfamethoxazole)	Ciprofloxacin 20 mg/kg/d for 3 d Ceftriaxone IV at 50–100 mg/kg per day for 5–7 d
Suspected cholera (age ≥2 y + severe dehydration + cholera present in area)	Erythromycin (12 mg/kg) 4 times a day for 3 d Ciprofloxacin 10–20 mg/kg twice per day for 5 d Trimethoprim-sulfamethoxazole: 4 mg/kg trimethoprim & 20 mg/kg sulfamethoxazole twice a day	Not in the national guideline Reference to WHO guideline

Abbreviations: IM, intramuscular; IV, intravenous; ORS, oral rehydration solution; WHO, World Health Organization.

## EPIDEMIOLOGY, CLINICAL PRESENTATION, AND MICROBIOLOGIC FEATURES OF *SHIGELLA*

In GEMS, it was shown that overall *Shigella* spp. accounted for one of the highest adjusted attributable fractions of moderate to severe diarrhea (MSD), with the highest attribution found in 12- to 23-month-olds. Moreover, among 25- to 59-month-olds, the highest attributable fraction was due to *Shigella* [10]. The attributable fraction due to *Shigella* detected in culture was 2.4% and 2.0% in Mali in 12- to 23-month-olds and 24- to 59-month-olds, respectively [17]. When samples were analyzed using quantitative polymerase chain reaction, the attributable fraction increased to 4%, 27.1%, and 20.1% in 0- to 11-month-olds, 12- to 23-month-olds, and 25- to 59-month-olds, respectively, confirming that it was also an important pathogen in Bamako, Mali [18]. *Shigella* also had the highest adjusted attributable fraction of antibiotic-treated diarrhea among 24- to 59-month-olds (11%) and was second highest in 12- to 23-month-olds (21.3%) [19].

In VIDA, after the introduction of rotavirus vaccine in 2014, *Shigella* had an episode-specific attributable fraction of 9.3% in Mali, with most cases occurring in 12- to 23-month-olds [15]. The children typically presented with watery diarrhea (84.6%, 86.5%, and 77.1% in infants [0–11 months], toddlers [12–24 months], and young children [25–59 months], respectively). Cases peaked from June to September, during the rainy season. Most cases were *S. flexneri*, serotypes 2a and X; *S. sonnei* and *S. boydii* were also observed [15]. Antibiotic resistance testing, though limited by the number of isolates, demonstrated 100% resistance to cotrimoxazole and 100% susceptibility to azithromycin, ceftriaxone, and ciprofloxacin.

## LOCAL HEALTH CARE–SEEKING

In Mali, the first point of contact for children with diarrhea is most commonly the traditional healer (52.3%) [5]. This preference is largely driven by cost as they charge less than the local health centers. They prescribe herbal medicines that they have made and that differ in content between healers. These may contain antibiotics, but this is not standardized. In GEMS and VIDA, CVD-Mali worked with the traditional healers and asked them to refer participants to participating health centers for inclusion in the studies. They accepted this practice, and the study team enrolled participants and recommended management according to the national guidelines. This referral system will not be used in EFGH.

## REGULATORY APPROVALS

Before undertaking study activities, the protocol and supporting documents will be approved by the local Ethics Committee at the University of Science, Techniques and Technology of Bamako, the University of Maryland Institutional Review Board, and the University of Washington.

## TRAINING AND CAPACITY BUILDING

Mali is a land-locked, francophone country ranked by the United Nations in 2021 as having the sixth lowest human development index and the seventh highest under-5 mortality rate [20]. Landlocked countries in Africa face immense challenges to socioeconomic development; they depend on neighboring countries that have maritime borders for transportation of goods, which can lead to delays, higher costs, and ultimately the stagnation of economies, with limited opportunities to develop exportable manufacturing infrastructure. Fragile economies fuel political instability. Desertification in Mali threatens agriculture and livestock productivity. Isolation diminishes access to educational resources, including underexposure to scientific literature, which is overwhelmingly in English, and to scientific conferences. The collaboration between the Malian Ministry of Health and University of Maryland School of Medicine since 2002 has addressed equity, diversity, and inclusion by creating educational opportunities and generating data that lead to implementing of public health measures for women and children such as vaccines, building clinical, laboratory, and bioinformatic infrastructure, and technology transfer. In VIDA, as an example, we initiated an iterative structured process for local site investigators to lead the writing of manuscripts for a journal supplement, posing scientific questions, formulating statistical analysis plans, writing manuscripts, and assisting with the manuscript submission process.

## CONCLUSIONS

Since 2007, CVD-Mali has developed an infrastructure that enables detailed investigations of the incidence, etiology, and clinical sequelae of diarrheal diseases among infants and young children in Mali to inform the development of effective interventions. GEMS and VIDA have shown the importance of *Shigella* as a cause of acute illness and longer-term mortality and nutritional sequelae and have characterized the serotypes important for inclusion in a vaccine and the age groups with the greatest burden [15, 21–24]. Improving adherence to IMCI-recommended management of diarrhea, including use of fluids, electrolytes, and zinc, continues to be a challenge. Given the clinical and epidemiologic burden, combined with the frequent use of antibiotics for diarrheal diseases for which there is no indication and the rising threat of multiresistant *Shigella*, there appears to be a need for a safe and effective vaccine. As results from phase 1 and 2 clinical trials in endemic settings come to fruition, results from EFGH will inform the conduct trials with promising candidates.
